# Metastatic Pheochromocytoma in a Patient With Li-Fraumeni Syndrome

**DOI:** 10.1210/jcemcr/luaf166

**Published:** 2025-08-01

**Authors:** Sotiris Loizidis, Christiana Matthaiou, Efrosini Iacovou, Karel Pacak, Ashley Grossman

**Affiliations:** Medical Oncology Department, Bank of Cyprus Oncology Center, Nicosia 2006, Cyprus; Medical Oncology Department, Bank of Cyprus Oncology Center, Nicosia 2006, Cyprus; Independent Histopathology Services, ECCLabs-IHCS, Nicosia 2020, Cyprus; Center for Adrenal Endocrine Tumors, AKESO, Prague 155 00, Czech Republic; Section on Medical Neuroendocrinology, Eunice Kennedy Shriver National Institute of Child Health and Human Development, National Institutes of Health, Rockville, MD 20847, USA; Centre for Endocrinology, Barts and the London School of Medicine and Dentistry, Queen Mary University of London, London EC1M 6BQ, UK; Green Templeton College, University of Oxford, Oxford OX2 6HG, UK; ENETS Centre of Excellence, Royal Free Hospital, London NW3 2QG, UK

**Keywords:** pheochromocytoma, paraganglioma, Li-Fraumeni syndrome, genetics, P53, molecular clusters

## Abstract

Li-Fraumeni syndrome (LFS) is a rare cancer predisposition syndrome caused by genomic alterations in the tumor protein p53 (*TP53*) gene. The lifetime risk of developing cancer is very high, and carriers of germline *TP53* pathogenic variants must be closely monitored starting from a young age. LFS is particularly associated with specific tumors, such as breast cancer, soft tissue and bone sarcomas, primary central nervous system cancers, acute leukemia, and adrenocortical carcinoma. Despite its association with a broad spectrum of malignancies, pheochromocytoma/paraganglioma (PCC/PGL) is an unusual manifestation of LFS and has only rarely been reported. Here, we present a case of a 57-year-old female patient who is a carrier of a deleterious germline *TP53* pathogenic variant and developed a PCC; several years later, she had lung and bone lesions compatible with metastatic PCC. We also discuss the most recent literature regarding the genomic landscape of PCCs/PGLs and their pathogenesis in connection with *TP53* pathogenic variants.

## Introduction

Li-Fraumeni syndrome (LFS) is a rare inherited cancer syndrome with an autosomal hereditary pattern. It was first described in 1969, while approximately 20 years later, germline tumor protein p53 (*TP53*) pathogenic variants were established as the cause of the syndrome [1, 2]. Individuals with LFS bear a markedly increased lifetime risk for neoplasms such as early-onset breast cancer, soft tissue and bone sarcomas, primary central nervous system cancers, acute leukemia and adrenocortical carcinoma, requiring early and strict lifetime surveillance [3]. The *TP53* gene, often referred to as the “guardian of the genome,” is dormant under physiological conditions. Cellular stress signals enable the binding of p53 protein to DNA, activating genes responsible for DNA repair, cycle arrest, and apoptosis, aiming to restore normality [4]. This safety brake is abolished by malfunctioning forms of the p53 protein, rendering cells highly vulnerable to DNA damage, ultimately leading to a cascade of tumorigenic events. Most LFS cases are inherited in an autosomal-dominant pattern; however, 7% to 20% of cases are attributed to de novo mutations or mosaicism [5]. Developing a pheochromocytoma/paraganglioma (PCC/PGL) represents a rather unusual presentation in *TP53* carriers. We report a female patient with a germline *TP53* pathogenic variant who developed metastatic PCC, and we discuss the current literature on this rare situation.

## Case Presentation

A 57-year-old woman with LFS was under surveillance at our center. Her past medical history was remarkable for type 2 diabetes mellitus, hypertension, and various malignancies. In 2009, she was diagnosed with papillary thyroid cancer (pT1bN0) and treated with near-total thyroidectomy followed by radioactive iodine therapy. In 2014, a 10-cm asymptomatic adrenal mass was incidentally discovered. Magnetic resonance imaging (MRI) of the abdomen suggested a pheochromocytoma, while suspicious para-aortic lymphadenopathy was noted. The preoperative hormonal profile for catecholamine hypersecretion was negative. The patient underwent surgery, revealing a 9-cm well-circumscribed mass composed of large polygonal cells arranged in nests (Zellballen pattern), a low proliferation index (Ki-67 = 1%-3%), rare mitoses (<2 mitoses/10 high-power fields), absence of necrosis, and low cellularity. Three lymph nodes were negative for metastases. Immunohistochemistry (IHC) was positive for synaptophysin, chromogranin A, S100, and GATA-binding protein 3 (GATA3). The pathology features were consistent with PCC (pT2N0). The Pheochromocytoma of the Adrenal gland Scaled Score (PASS) was 2, and the Grading system for Adrenal Pheochromocytoma and Paraganglioma (GAPP) had a score of 1, both indicating a low probability for metastasis. In 2015, she was diagnosed with high-grade ductal carcinoma in situ (DCIS) of the left breast. She underwent a partial mastectomy followed by radiation therapy and initiated adjuvant endocrine treatment with tamoxifen. A few months later, multiple lesions were identified in the left breast, and a subsequent biopsy showed an invasive breast carcinoma (triple-negative breast cancer). She underwent a left mastectomy and axillary lymph node removal [pT2(m)N1] followed by adjuvant chemotherapy. The presence of multiple malignancies prompted targeted genetic testing (Sanger sequencing) for *BRCA1/2* and *TP53* gene alterations. Testing revealed a substitution in exon 6 (c.743G>A) of the *TP53* gene, resulting in arginine substitution by glutamine at codon 248 (p.Arg248Gln). This missense mutation represents an established pathogenic variant, and the patient was placed on a surveillance program [6]. In 2018, she underwent a prophylactic right mastectomy. Her daughter and 2 sisters tested negative for the *TP53* mutation, while her parents have not been assessed due to their advanced age. Her family history includes only one cancer case: her niece, with an unknown genetic background, was diagnosed with dermatofibrosarcoma protuberans.

## Diagnostic Assessment

**Figure 1.**
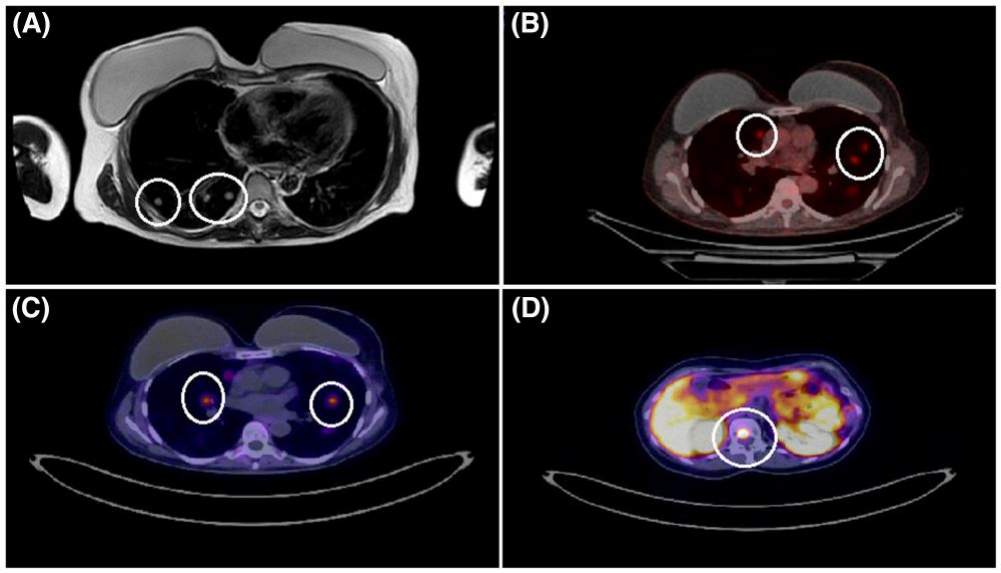
Imaging techniques were used during the case investigation. A. MRI of the abdomen (T2-weighted axial view) capturing the lower lung fields showed suspicious lesions (white circles). B. Fluorine-18 fluorodeoxyglucose PET/CT scan (axial view) depicted bilateral lung nodules (white circles). C. ^68^Ga-DOTATATE PET/CT scan (axial view) revealed somatostatin receptor-expressing lung lesions (white circles). D. SSTR-expressing tissue was also detected in the body of L1 by ^68^Ga-DOTATATE PET/CT scanning (axial view, white circle).

**Figure 2.**
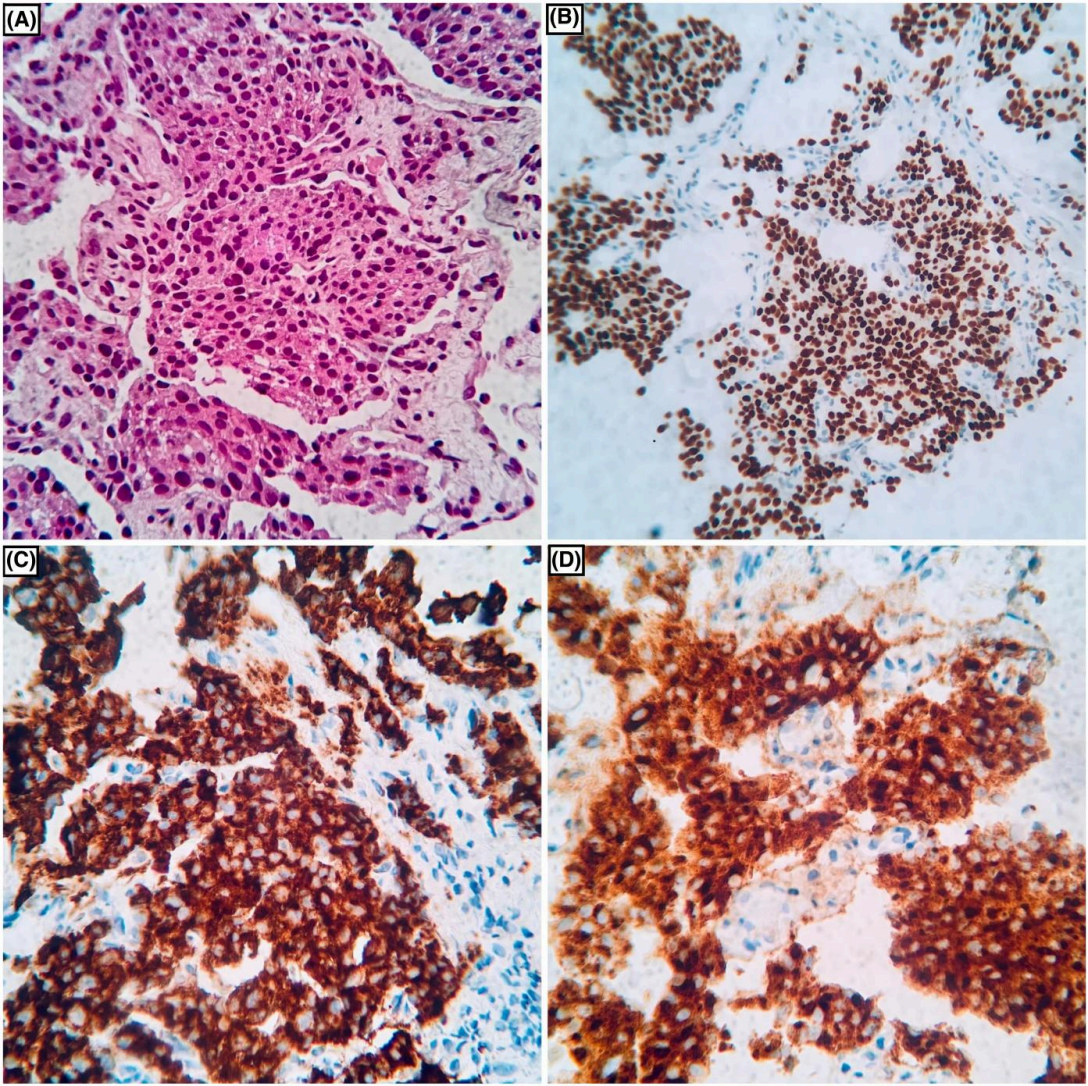
Histopathology images of the lung lesion. (A) Tumor cells arranged in nests (Zellballen pattern), hematoxylin and eosin-stained section at 200×. (B) Tumor cells showed strong GATA3 positivity. (C) Synaptophysin and (D) Chromogranin A staining are diffusely positive.

In 2023, her annual whole-body MRI (WBMRI) showed suspicious lung lesions warranting further investigation (Fig. 1A). A subsequent fluorine-18 fluorodeoxyglucose (^18^F-FDG) positron emission tomography/computer tomography (PET/CT) scan revealed multiple bilateral lung nodules (Fig. 1B). Following the imaging results, a CT-guided biopsy demonstrated infiltration by nests of tumor cells with eosinophilic cytoplasm and no mitotic activity. The IHC was positive for GATA3 and the neuroendocrine markers chromogranin A and synaptophysin, and negative for cytokeratin 7 (CK7), anti-cytokeratin 5.2 (CAM 5.2), cytokeratin AE1/3, cytokeratin 5/6 (CK5/6), thyroid transcription factor 1 (TTF-1), tumor protein 63 (p63), and estrogen receptor (ER). These findings were compatible with metastatic PCC (Fig. 2). Given the neuroendocrine nature of the neoplasm, a gallium-68 1,4,7,10-tetraazacyclododecane-1,4,7,10-tetraacetic acid (DOTA)–octreotate (^68^Ga-DOTATATE) PET/CT scan was performed. It confirmed the presence of somatostatin receptor-expressing tumor tissue in the lungs and in the first lumbar vertebrae (L1) (Fig. 1C and 1D). Clinically, the patient did not report any signs or symptoms due to excessive catecholamine secretion. A 24-hour urine collection for fractionated metanephrine, normetanephrine and 3-methoxytyramine was ordered, and the results were within normal limits (Table 1). Moreover, new germline testing was performed using a next-generation sequencing (NGS) assay (NextSeq 2000, Illumina, Inc.), interrogating a broad spectrum of genes related to PCCs/PGLs (*EGLN1, FH, MAX, MEN1, NF1, RET, SDHA, SDHA2, SDHB, SDHC, SDHD, TMEM127, VHL*). Testing was negative for any germline mutations other than *TP53*. The tissue sample was tested for additional somatic mutations (NextSeq 2000, Illumina, Inc.) and copy number variations (MLPA®, MRC Holland) with negative results.

**Table 1.** Results of the three 24-hour urine collections

Urine catecholamines	Results (SI units)	Normal range (SI units)
**First 24-hour urine collection (November 2023)**		
Metanephrine	62 μg/24 hours (314.3 nmol/day)	45-290 μg/24 hours (228.1-1470.3 nmol/day)
Normetanephrine	112 μg/24 hours (611.3 nmol/day)	75-400 μg/24 hours (409.3-2183.2 nmol/day)
Dopamine	68 μg/24 hours (443.9 nmol/day)	65-400 μg/24 hours (424.3-2611.2 nmol/day
**Second 24-hour urine collection (May 2024)**		
Metanephrine	191 μg/24 hours (968.4 nmol/day)	45-290 μg/24 hours (228.1-1470.3 nmol/day)
Normetanephrine	*^a^* **913 μg/24 hours (4983.1 nmol/day)**	75-400 μg/24 hours (409.3-2183.2 nmol/day)
Dopamine	73 μg/24 hours (476.5 nmol/day)	65-400 μg/24 hours (424.3-2611.2 nmol/day)
**Third 24-hour urine collection (October 2024)**		
Metanephrine	*^a^* **522 μg/24 hours (2646.5 nmol/day)**	45-290 μg/24 hours (228.1-1470.3 nmol/day)
Normetanephrine	*^a^* **1600 μg/24 hours (8732.8 nmol/day)**	75-400 μg/24 hours (409.3-2183.2 nmol/day)

Abbreviation: SI, Système International.

^*a*^Abnormal values are shown in bold font.

## Treatment

Given the absence of symptoms and signs and no evidence of catecholamine overproduction, the patient continued with follow-up and strict surveillance. A few months later, a new 24-hour urine test showed an increase in metanephrine levels, leading to the decision to start the patient on subcutaneous lanreotide autogel, 120 mg monthly. A WBMRI did not demonstrate any radiological disease progression. Subsequent repeat testing indicated a further rise in 24-hour urinary metanephrine levels, accompanied by blood pressure dysregulation. Antihypertensive therapy was modified to include doxazosin, 2 mg daily.

## Outcome and Follow-Up

The patient continues to be closely monitored for signs and symptoms of catecholamine excess. At present, she is asymptomatic, and her blood pressure is well-controlled. She undergoes regular WBMRI scanning; in due course, ^68^Ga-DOTATATE PET/CT will be repeated. In case of disease progression, further treatment options include radioisotope therapy with peptide receptor radionuclide therapy (PRRT) or iodine-131 meta-iodobenzylguanidine (^131^I-MIBG), systemic chemotherapy (cyclophosphamide-vincristine-dacarbazine, or temozolomide monotherapy), and molecular targeted therapy (cabozantinib, sunitinib).

## Discussion

PCCs/PGLs are rare neuroendocrine tumors arising from the adrenal medulla or extra-adrenal paraganglia. They are increasingly being discovered incidentally and predominantly produce catecholamines responsible for their symptomatology [7]. According to the latest World Health Organization (WHO) classification, virtually all PCCs/PGLs have metastatic potential, and the terms *benign* and *malignant* are no longer advised [8].

PCCs/PGSs have one of the highest genetic predispositions among all tumors, and international guidelines strongly endorse genetic testing. Approximately 30% to 40% of cases are associated with inherited syndromes harboring a germline mutation in genes related to PCCs/PGLs, while an additional 35% to 40% of tumors bear a somatic driver mutation [9]. Intriguingly, in 10% to 13% of seemingly sporadic cases, a germline pathogenic mutation in a culprit genetic locus is detected [10, 11]. Recent advances in molecular biology have enabled the in-depth characterization of the molecular background of these neoplasms. PCCs/PGLs can be assigned to 1 of 3 clusters based on the molecular pathway of tumorigenesis. Cluster 1, or the pseudo-hypoxic cluster, comprises genes that activate pathways resembling hypoxia signaling: these genes include *SDHA-D, FH, MDH2, SLC25A11,* and *VHL,* and are predominantly correlated with germline mutations. Cluster 2 includes genes implicated in tyrosine kinase signaling, such as *RET, HRAS, NF1, TMEM127, MAX, and FGFR1*. Mutations for some genes could be either germline or somatic, while for others, they are exclusively somatic. Cluster 3 is related to β-catenin/Wnt-signaling activation and comprises the *MAML3* fusion gene and *CSDE1*; mutations in this group are solely somatic [12].

The genetic landscape of PCCs/PGLs has largely been deciphered, but associations with *TP53* mutations have not been frequently observed. LFS appears to be strongly related to numerous types of cancer; however, the connection with PCCs/PGLs is infrequent and is only noted in sporadic case reports (Table 2). In 2016, Hu et al reported the first Chinese family with LFS, where one of the affected members was diagnosed with PCC at the age of 3 years [13]. A case of metastatic PCC in the context of LFS was reported by Gniado et al in 2021; however, the authors noted that the patient also carried another germline mutation in the *SDHB* gene, which is well known to be associated with PCC [14]. Seo et al reported a single *TP53* germline mutation in 36 patients with PCCs/PGLs [15]. Two cases of germline *TP53* mutations were reported in a North Korean series of 15 cases [16]. In a series of 101 PCCs/PGLs reported by Lima et al, there was one case of a germline *TP53* carrier, but the authors stated that the patient also had a germline pathogenic mutation in the *TMEM127* gene [17]. A case from Serbia was published regarding a carrier of a germline *TP53* pathogenic variant with a history of PCC [18].

**Table 2.** Cases of individuals with LFS who developed PCCs/PGLs

First author, year of publication [reference]	Demographics: age, gender	Type (PCC/PGL), stage	Catecholamine phenotype	TP53 mutation, classification
Hu et al., 2016 [13]	3 yo, female	PCC, local	NA	c.730G>A; p. (Gly244Ser), exon 6, PV
Gniado et al., 2020 [14]	39 yo, male	PCC, metastatic	NMN + MN	c.743G>A; p. (Arg248Gln), exon 6, PV
Seo et al., 2020 [15]	43 yo, female	PGL, local	NMN + MN	c.566C>T; p. (Ala189Val), exon 6, VUS
Choi et al., 2021 [16]	30 yo, female	PGL, metastatic	Functional tumor	c.725G>A; p. (Cys247Tyr), exon 7, PV
Choi et al., 2021 [16]	49 yo, female	PCC, metastatic	Functional tumor	c.31G>C p. (Glu11Gln), exon 18, VUS
Lima et al., 2023 [17]	32 yo, female	PCC, local	NMN + MN	c.1010G>A; p. (Arg337His), exon 9, PV/LP
Stojiljkovic et al., 2024 [18]	19 yo, female	PCC, local	Functional tumor	c.376-2A>G, intronic region (splice acceptor), PV/LP

Abbreviations: LP, likely pathogenic; MN, metanephrine; NA, not applicable; NMN, normetanephrine; PCC/PGL, pheochromocytoma/paraganglioma; PV, pathogenic variant; VUS, variant of undetermined significance.

As noted above, extensive molecular profiling has greatly illuminated the genomic blueprint of PCCs/PGLs. Although the loss of the short arm of chromosome 17, which contains the genetic locus of *TP53*, is considered a frequent genomic event in PCCs/PGLs, more specific *TP53* genomic alterations have also been identified [10, 19]. The French COMETE cohort was one of the first initiatives aiming to shed light on the genomic landscape of PCCs/PGLs. A multi-omics analysis of 202 samples revealed somatic mutations in the *TP53* gene in only 2 cases [20]. Another initiative was launched by the Cancer Genome Atlas (TCGA) research network by conducting a comprehensive molecular characterization of 173 PCCs/PGLs. No germline *TP53* germline pathogenic variants were detected, while somatic alterations were found in just one case [10]. A large cohort conducted in the United Kingdom that included 141 individuals with PCCs/PGLs aimed to investigate the clinical utility of somatic sequencing: 45 patients harbored germline mutations, but none involving the *TP53* gene, and while 37 had pathogenetic somatic mutations, only one referred to *TP53* [21]. Other smaller studies have confirmed the rarity of germline and somatic *TP53* mutations [16, 22-24].

Searching the literature, we detected 7 germline *TP53* variants (0.74%) in 942 PCCs/PGLs with germline investigation [10, 13-18, 20-24], while of 788 cases with somatic testing, 10 bore somatic variants (1.27%) [10, 16, 20-24]. Table 3 summarizes the characteristics of PCCs/PGLs with *TP53* alterations. Most carriers with germline mutations are female and tend to develop PCCs (71.4%), which are functional tumors (100%) with mixed catecholamine secretion profiles. When metastatic, the tumors tended to metastasize primarily to bones and lungs. In 2 cases, germline co-mutations with *SDHB* and *TMEM127* genes were detected. Both genes are related to the development of PCCs/PGLs, and the contribution of the *TP53* mutation to tumorigenesis in this context is unclear.

**Table 3.** Characteristics of PCCs/PGLs with TP53 germline and somatic mutations

	Number [ref.]	Gender (M/F)	Median age (range)	Type (PCC/PGL) (%/%)	Local/metastatic disease (%)	Metastatic sites	Functional status (%)	Other co-mutations (germline/somatic)
**Somatic**	*^a^* 10 [10, 16, 20-24]	2/6	42 (25-66)	7/3 (70/30)	L:5 (50) M:3(30)	Kidneys, LNs	4/10 (40)	1 *RET*→G (VUS) 3 *SDHB*→G (1 NA, 2 PV) 1 *NF1*→S
**Germline**	*^a^* 7 [13-18]	1/6	37 (3-49)	5/2 (71.4/28.6)	L:4 (57.1) M:3(42.9)	Bones, lungs, LNs	6/6 (100)	1 *NF2*→G (BV) 1 *SDHB*→G (PV) 1 *TMEM127*→G (PV) 1 *HRAS*→S 1 *NF1*→S 1 *SDHB*→S

Abbreviations: BV, benign variant; F, female; G, germline; L, local; LNs, lymph nodes; M, male; M, metastatic; NA, not applicable; PCC/PGL, pheochromocytoma/paraganglioma; PV, pathogenic variant; S, somatic; VUS, variant of undetermined significance.

^*a*^Data for some cases were inadequate.

In our case, the knockout of the wild-type *TP53* allele, leading to loss of heterozygosity, could represent a potential tumorigenesis mechanism. However, molecular investigation of the tumor did not reveal a deleterious mutation or loss of the genetic locus of the wild-type *TP53* gene; thus, epigenetic silencing of the wild-type *TP53* allele could be speculated as a possible mechanism. Geli et al showed that PCCs/PGLs exhibit an extensive epigenomic dysregulation, leading to epigenetic silencing of tumor suppressor genes and correlating with aggressive disease [25]. Furthermore, mutations in genes related to epigenetic regulation, such as *ATRX, SETD2,* and *KMT2D*, have been described in PCCs/PGLs [12]. It has also been demonstrated that PCCs/PGLs with gene mutations included in Cluster 1 exhibit a profound hypermethylation phenotype [26]. Another theory supports the concept that deleterious germline *TP53* mutations exert a predominantly dominant-negative effect on the wild-type *TP53* allele, abrogating normal protein function [27].

Our patient was exposed to radiation following the diagnosis of DCIS, and a few months later she developed invasive carcinoma. Although the evidence regarding the impact of radiation on *TP53* carriers is scarce, radiotherapy appears to further increase the risk of secondary malignancies, particularly for soft tissue sarcomas [28]. PRRT is an established treatment for PCCs/PGLs. Typically, the recommended schedule for PRRT with lutetium-177 (^177^Lu)-DOTATATE consists of 4 fixed cycles of 7.4 GBq (200 mCi) infusions every 6 to 12 weeks. Nevertheless, the effects of the emitted radiation from this type of therapy in LFS individuals remain unknown, and its possible future use in this patient would need to be more carefully considered than in patients with other types of mutation.

## Learning Points

- LFS is associated with a broad spectrum of malignancies. PCCs/PGLs represent infrequent manifestations of the syndrome (≈1%).
- Carriers of deleterious germline *TP53* variants should be closely monitored from a young age. Screening recommendations propose comprehensive physical examination, ultrasound of the abdomen and pelvis every 3 to 4 months, and annual WB-MRI for carriers starting at birth.
- Based on the existing literature, we recommend periodic blood pressure monitoring and consideration of assessments of metanephrines secretion wherever there is clinical suspicion. Where positive, one could proceed to careful evaluation of WBMRI for adrenal lesions and ^68^Ga-DOTATATE PET/CT scanning may be useful.

## Contributors

All authors made individual contributions to authorship. S.L., C.M., and A.G. were involved in the diagnosis and management of this patient; E.I. was involved in histopathology pictures presentation; and K.P. and A.G. were involved in manuscript editing and correction. All authors reviewed and approved the final draft.

## Data Availability

Data sharing is not applicable to this article as no datasets were generated or analyzed during the current study.

## Abbreviations

DOTATATE: 1,4,7,10-tetraazacyclododecane-1,4,7,10-tetraacetic acid (DOTA)–octreotate
GATA3: GATA-binding protein 3
IHC: immunohistochemistry
LFS: Li-Fraumeni syndrome
MRI: magnetic resonance imaging
PCC: pheochromocytoma
PET/CT: positron emission tomography/computer tomography
PGL: paraganglioma
PRRT: peptide receptor radionuclide therapy
WBMRI: whole-body magnetic resonance imaging
